# Epigenetic alterations in the placenta associated with SARS-CoV-2 infection during pregnancy

**DOI:** 10.1007/s00404-026-08589-8

**Published:** 2026-11-13

**Authors:** Franziska Ganster, Friederike Kallenberg, Anna Titova, Laura Hahn, Susanne Beyer, Maximilian Stocker, Maria Fischer, Mirjana Kessler, Sven Mahner, Thomas Kolben, Udo Jeschke, Sarah Meister

**Affiliations:** 1 Department of Obstetrics and Gynecology, University Hospital, LMU Munich, Marchioninistr. 15, 81377 Munich, Germany; 2 Department of Medicine I, University Hospital Munich, Ludwig-Maximilian-University Munich (LMU), Munich, Germany; 3 Institute of Surgical Research at the Walter Brendel Center of Experimental Medicine, University Hospital Munich, Ludwig-Maximilians-University (LMU), Munich, Germany; 4 Walter Brendel Center of Experimental Medicine, University Hospital Munich, Ludwig-Maximilians-University (LMU), Munich, Germany; 5 Department of Gynecology and Obstetrics, University Hospital Augsburg, Stenglinstr. 2, 86156 Augsburg, Germany

**Keywords:** Histone modifications, SARS-CoV-2 infection, Placental function, H3K4me3 and H3K9ac, Epigenetics in pregnancy

## Abstract

Histone modifications play a key role in chromatin organization and transcriptional regulatory potential. Previously, reduced levels of trimethylated histone H3K4me3 and acetylated H3K9ac were observed in placentas from women with preeclampsia. Building on these findings, this study investigated whether SARS-CoV-2 infection during pregnancy or at delivery is associated with changes in placental histone modifications. Immunohistochemical staining, exploratory western blot analysis, and qualitative immunofluorescence were performed on placental sections from 60 patients stratified into four groups: women with acute SARS-CoV-2 infection at delivery, women with prior infection during gestation, vaccinated controls, and unvaccinated controls. H3K4me3 levels in the syncytiotrophoblast were significantly reduced in both SARS-CoV-2-exposed groups compared to unvaccinated controls, with the most pronounced reduction observed in women with prior infection during gestation. H3K9ac levels did not differ significantly between groups in any compartment after correction for multiple comparisons. No significant associations were found between SCT H3K4me3 levels and maternal age, gestational age, or birthweight, and a sensitivity analysis excluding cases with additional placental pathologies confirmed the robustness of the findings. As no gene expression profiling was performed, functional or transcriptional consequences of the observed H3K4me3 changes cannot be inferred. These descriptive findings warrant further investigation integrating epigenetic and transcriptomic approaches to clarify their functional relevance for placental biology and pregnancy outcomes.

## What does this study add to the clinical work

**Table:** 

Our findings demonstrate an association between SARS-CoV-2 infection during pregnancy and reduced H3K4me3 levels in the syncytiotrophoblast. These results provide a foundation for future studies integrating epigenetic analyses.	

## Introduction

The emergence of severe acute respiratory syndrome coronavirus 2 (SARS-CoV-2), responsible for the coronavirus disease 2019 (COVID-19) pandemic, has brought unprecedented challenges to global healthcare systems [1]. While much attention has been directed towards understanding the clinical manifestations and epidemiology of COVID-19 in the general population [2–4], there is a pressing need to elucidate its effects on vulnerable populations, particularly pregnant individuals and their developing fetuses.

Pregnancy represents a unique immunological state characterized by profound physiological changes that modulate maternal immune responses to accommodate the semi-allogeneic fetus [5, 6]. Emerging evidence suggests that pregnant individuals may experience altered immune responses, potentially rendering them more susceptible to severe outcomes upon SARS-CoV-2 infection [7, 8]. Moreover, concerns have been raised regarding the potential vertical transmission of the virus from mother to fetus, highlighting the importance of understanding the impact of maternal infection on placental health and fetal development [9–14].

Epigenetic mechanisms, including histone modifications, play crucial roles in regulating gene expression and chromatin structure during pregnancy [15, 16]. Among these modifications, trimethylation of histone H3 at lysine 4 (H3K4me3) and acetylation at lysine 9 (H3K9ac) have been studied in the context of placental development, trophoblast differentiation, and maternal–fetal communication [17, 18]. In a previous study, we demonstrated that H3K4me3 and H3K9ac levels are significantly reduced in placentas from individuals with preeclampsia compared to normotensive controls, suggesting a potential link between epigenetic alterations and the pathophysiology of this pregnancy complication [19, 20]. Building on these findings, the present study investigates whether maternal SARS-CoV-2 infection is associated with changes in placental H3K4me3 and H3K9ac. Given the pathophysiological overlap between preeclampsia and COVID-19-related pregnancy complications including endothelial dysfunction and inflammation, we hypothesized that SARS-CoV-2 infection may induce similar epigenetic changes in placental tissue. To address this, immunohistochemical staining alongside exploratory western blot and immunofluorescence analyses were performed on placental sections from four groups: women with acute SARS-CoV-2 infection at delivery, women with prior infection during gestation, vaccinated controls, and unvaccinated controls.

## Materials and methods

### Sample Collection

Placental samples were collected at the Department of Obstetrics and Gynecology, LMU University Hospital Munich, between August 2017 and July 2022. The cohort was stratified into four groups: women who had received SARS-CoV-2 vaccination and never knowingly had an infection (*n* = 13), and women who had neither been infected nor vaccinated, whose tissue was collected prior to the pandemic (*n* = 16). The two groups of interest consisted of women with acute SARS-CoV-2 infection at delivery (*n* = 15) and women with a prior infection during gestation (*n* = 16) (Table 1). No distinction was made between available vaccine types. The unvaccinated, uninfected control samples were collected prior to the onset of the COVID-19 pandemic (August 2017 to February 2020), whereas samples from the SARS-CoV-2-exposed and vaccinated groups were collected during the subsequent pandemic period (March 2020 to July 2022). Acute SARS-CoV-2 infection at delivery was ascertained by positive PCR or rapid antigen test at the time of admission. Prior infection during gestation was ascertained based on documented positive test results recorded in the patients' medical files. Following written informed consent, placental samples were collected promptly postpartum and processed according to the same study protocol. One portion was fixed in 4% formalin and subsequently paraffin-embedded for immunohistochemistry and immunofluorescence. All samples were processed, stored, and analyzed under identical experimental conditions using the same protocols and reagents.

**Table 1:** Patient characteristics across the four study groups

	Maternal age			
P value	0.4441			
	Control no vaccine	Control vaccine	COVID	S/P COVID
Number of values	16	13	15	16
Minimum	23	26	20	25
Maximum	37	37	42	37
Range	14	11	22	12
Mean	31.88	33.23	31.87	31.25
Std. deviation	4.08	3.244	5.54	3.215
Std. error of mean	1.02	0.8998	1.431	0.8036

	Birth weight in grams			
P value	0.4193			
	Control no vaccine	Control vaccine	COVID	S/P COVID
Number of values	16	13	15	16
Minimum	2550	2500	1790	2360
Maximum	4520	4350	4130	4245
Range	1970	1850	3340	1885
Mean	3461	3496	3070	3364
Std. deviation	551.6	457.2	858.8	466.2
Std. error of mean	137.9	126.8	221.7	116.5

	Gestational age at birth in days			
P value	0.1737			
	Control no vaccine	Control vaccine	COVID	S/P COVID
Number of values	16	13	15	16
Minimum	251	260	175	251
Maximum	290	291	294	291
Range	39	31	119	40
Mean	274.4	278.3	262.2	275.6
Std. deviation	12.2	9.481	29.54	9.619
Std. error of mean	3.05	2.63	7.628	2.405

The final cohort comprised 60 participants, representing samples for which measurements of both H3K4me3 and H3K9ac were available, allowing complete analysis of both histone modifications.

Promptly postpartum, following written informed consent, placental samples were collected and divided; one portion was fixed in 4% formalin and subsequently paraffin-embedded for use in immunohistochemistry and immunofluorescence. The final cohort comprised 60 participants, as these were the samples for which both H3K4me3 and H3K9ac measurements were available, allowing for complete analysis of both histone modifications.

### Western blot

Western blot analysis was performed using frozen tissue samples from four experimental groups: post-SARS-CoV-2 during pregnancy, vaccinated control, unvaccinated control, and acute SARS-CoV-2 infection. Organs were stored at −80 °C and transferred into 2 mL Eppendorf tubes on ice. Each sample was processed in 0.4 mL of ice-cold protein preparation buffer (PPB; 50 mM HEPES, 330 mM sucrose, pH 7.4, 0.2 µm filtered) supplemented with 1× Complete™ protease inhibitor cocktail (Roche) and 2 µM sodium orthovanadate. The tissue was minced and homogenized on ice using an ultrasonic probe (4 × 20 s pulses at setting 3). Homogenates were transferred into fresh tubes and centrifuged at 20,000×*g* for 10 min at 4 °C. An aliquot of 30 µL from each lysate was reserved for protein concentration determination using the BCA assay. The remaining supernatant was mixed with 1× SDS sample buffer (62.5 mM Tris-HCl, pH 6.8; 2% SDS; 100 mM β-mercaptoethanol; a trace of bromophenol blue), boiled at 96 °C for 5 min, and either directly used for electrophoresis or stored at −20 °C.

Proteins were separated by SDS-PAGE using Precision Plus Protein™ All Blue Prestained Protein Standards (#1610373, Bio-Rad) and transferred onto PVDF membranes. The membranes were incubated with the following primary antibodies: anti-H3K4me3 (rabbit, ab8580, Abcam; 1:1000), anti-H3K9ac (rabbit, ab32129, Abcam; 1:500), and anti-β-actin (mouse, A5441, Sigma; 1:1000). HRP-conjugated secondary antibodies were used for detection: goat anti- mouse (G-21040, Invitrogen; 1:2000) and goat anti-rabbit (G-21234, Invitrogen; 1:5000). Chemiluminescent substrate was used for signal development and visualization.

### Immunohistochemical staining (IHC)

Employing established methodologies as previously described by [20, 21] immunohistochemical staining was carried out. Briefly, following multiple washing steps and antigen retrieval via citric acid solution, a blocking agent was used to prevent unspecific staining (Detection system ZytoChem Plus HRP polymer system (mouse/rabbit), Zytomed, nr. POLHRP-100). Next, incubation with primary antibodies (Anti-H3K9ac, Order no. Ab32129 Rabbit monoclonal, and Anti-H3K4me3, Order no. Ab8580 Rabbit polyclonal) followed for a duration of 16 h overnight. Post-incubation, a post-blocking step was carried out, followed by visualization of the staining using liquid chromogen 3,3′-Diaminobenzidine (Liquid DAB+ Substrate Chromogen System (Dako; Best.Nr. K3468) with an individual incubation period for each antibody. Lastly, counterstaining followed by putting the slides into Mayer’s hemalaun.

### Immunofluorescence staining

The immunofluorescence staining followed established protocols as described by [20, 21]. Immunofluorescence staining was performed in 3 samples per group for each histone modification. Initially the slides underwent deparaffinization in Roticlear, followed by rehydration in a descending alcohol series. Incubation overnight followed a blocking step to prevent unspecific staining. The next day, the slides were incubated with secondary antibodies functioning as the fluorochromes (AlexaFluorTM Plus 555, Donkey Anti-rabbit, Thermofisher A32794, CyTM5 AffiniPureTM, Donkey Anti-mouse, Jackson Immuno Research, Order-No 715-175-151). For nucleus staining 4′,6′-Diamino-2-phenylindole (DAPI) was used. We assessed the ratio of histone modification to nucleus.

### Staining evaluation

#### Staining evaluation via IRS

The semi-quantitative immunoreactive score (IRS) served as the evaluation tool, integrating the staining intensity and the proportion of positively stained cells. Intensity gradation (0 = no reaction, 1 = weak intensity, 2 = moderate intensity, 3 = high intensity) was multiplied by the percentage of positively stained cells (0 = none, 1 = less than 10%, 2 = 10–50%, 3 = 51–80%, 4 = more than 81%), thereby resulting in the IRS. Two scores were obtained for each slide—one for the fetal part of the placenta, the syncytiotrophoblasts, and two for the maternal part of the placenta, the decidua. Within the decidua the extravillous trophoblasts (EVT) and decidual stroma cells were evaluated. For each section, five high-power fields (HPF) per compartment were evaluated at 400× magnification. Fields were selected systematically by moving across the section in a defined pattern. IRS scoring was performed by a single observer blinded to group assignment. Inter-rater reliability assessment was not applicable as scoring was conducted by one observer throughout.

### Statistics

Statistical analyses were performed using GraphPad Prism (Version 10.5.0). As IRS values were ordinal and non-normally distributed, non-parametric tests were used throughout. Group differences across the four study groups were assessed using the Kruskal–Wallis test, followed by Dunn's post-hoc test with Bonferroni correction for multiple pairwise comparisons. Results are reported as median and interquartile range (IQR). Spearman rank correlation (Spearman-Rho) was used to examine associations between IRS scores and continuous clinical variables. The correlation coefficient r was interpreted as follows: r < 0.3 weak, r = 0.3–0.5 moderate, r > 0.5 strong. A p-value < 0.05 was considered statistically significant.

## Results

### Descriptive statistics

Table 1 summarizes the patient cohort. A total of 60 patients were enrolled across four groups: unvaccinated controls (n = 16), vaccinated controls (n = 13), patients with acute SARS-CoV-2 infection at delivery (COVID; n = 15), and patients with prior infection during gestation (S/P COVID; n = 16). Among the COVID group, four women experienced severe infections and two required intubation. The vaccinated control group was heterogeneous, including women who had received between one and four vaccine doses. In the S/P COVID group (n = 16), the majority of women (n = 12; 75%) had experienced infection before 34 weeks of gestation. Additional placental pathologies observed in the cohort included preeclampsia (n = 1, S/P COVID group), placenta previa (n = 1, S/P COVID group), and placenta accreta (n = 1, unvaccinated control group).

The four groups were comparable with respect to maternal age (mean 31.9 vs. 33.2 vs. 31.9 vs. 31.3 years; p = 0.444), birthweight (mean 3461 vs. 3496 vs. 3070 vs. 3364 g; p = 0.419), and gestational age at birth (mean 274 vs. 278 vs. 262 vs. 276 days; p = 0.174), with no statistically significant differences across groups (Table 1).

### H3K4me3 and H3K9ac in SARS-CoV-2-exposed placentas

#### Western blot of H3K4me3 and H3K9ac in COVID placentas

As an initial exploratory assessment of H3K4me3 and H3K9ac protein levels across the four groups, western blot analysis was performed on frozen placental tissue. Sample availability limited group sizes to n = 3 for the unvaccinated control, vaccinated control, and S/P COVID groups, and n = 2 for the acute COVID group. Given these small sample sizes and the high inter-sample variability observed, no formal statistical testing was performed; results are presented as illustrative only.

**Fig. 1:**
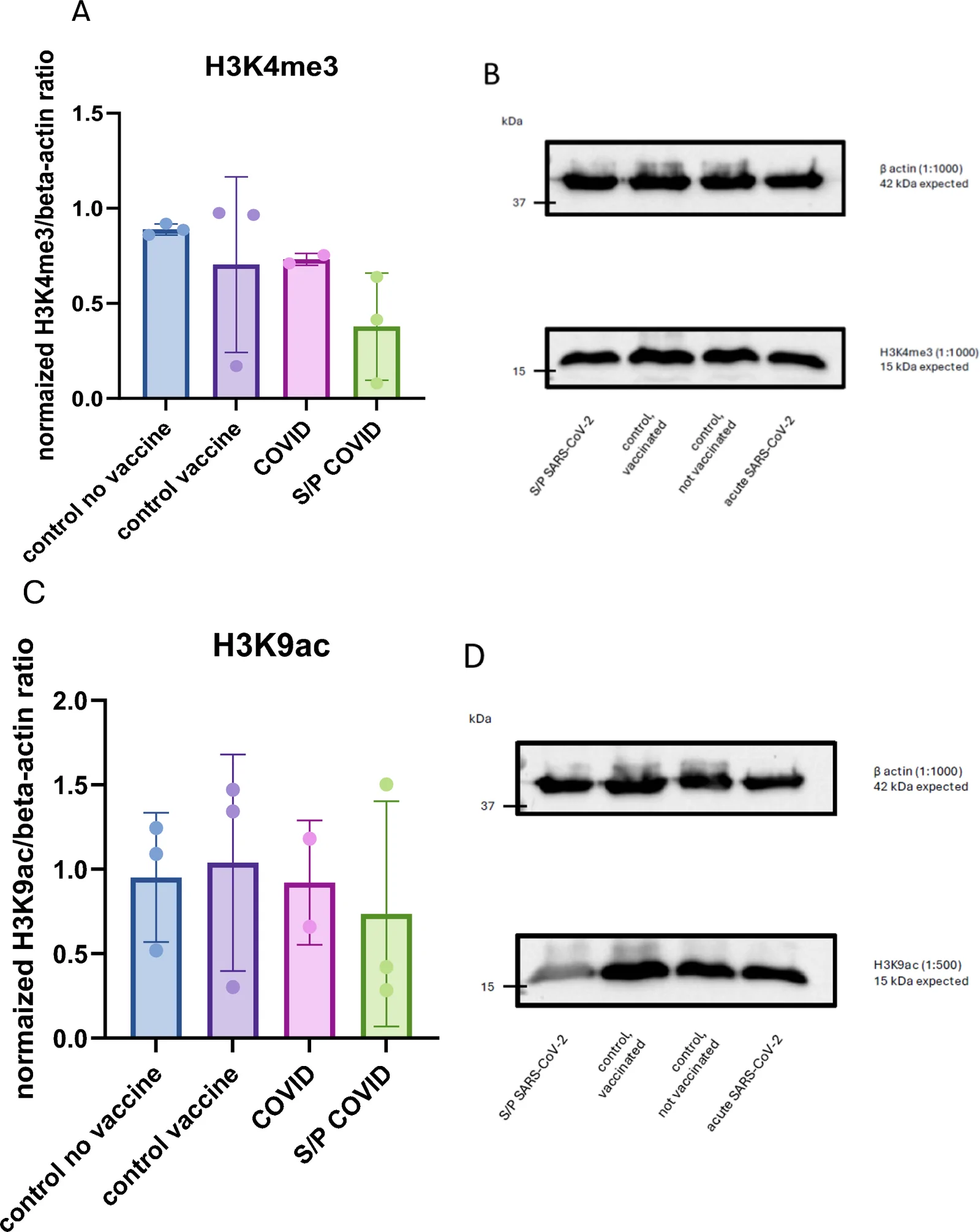
Exploratory western blot analysis of H3K4me3 (**A**, **B**) and H3K9ac (**C**, **D**) in frozen placental tissue. **A** ß-actin-normalized H3K4me3 values for unvaccinated controls, vaccinated controls, acute COVID, and S/P COVID groups. **B** Representative western blot protein bands for H3K4me3 and ß-actin. **C** ß-actin-normalized H3K9ac values. **D** Representative western blot protein bands for H3K9ac and ß-actin. Sample sizes: n = 3 for unvaccinated control, vaccinated control, and S/P COVID groups; n = 2 for the acute COVID group. Data points represent individual samples; bars indicate group means. Due to small sample sizes and high inter-sample variability, no statistical comparisons were performed; findings are exploratory and illustrative only

ß-actin-normalized H3K4me3 levels showed a trend toward reduction in the S/P COVID group (mean 0.378 ± 0.282) compared to unvaccinated controls (mean 0.889 ± 0.030). The acute COVID group showed an intermediate value (mean 0.732, n = 2). The vaccinated control group showed high inter-sample variability (mean 0.704 ± 0.462), driven by one outlier sample, and should be interpreted with caution (Fig. 1A, B).

For H3K9ac, no clear directional pattern was observed across groups. Mean normalized values were 0.951 ± 0.382 for unvaccinated controls, 1.039 ± 0.641 for vaccinated controls, 0.920 for the acute COVID group (n = 2), and 0.736 ± 0.667 for the S/P COVID group. All groups showed substantial inter-sample variability, precluding any meaningful interpretation of group differences at this sample size (Fig. 1C, D).

#### Trimethylated histone H3K4me3 reduced in SCT of COVID placentas

Placental sections from all four groups were stained with anti-H3K4me3 antibody, and IRS scores were determined separately for the syncytiotrophoblast (SCT), extravillous trophoblasts (EVT), and decidual stromal cells. Because IRS values showed a non-normal distribution with considerable inter-individual variability, results are reported as median (IQR); full descriptive statistics are provided in Table 2.

**Table 2:** Summary of IRS scores (median and IQR) of H3K4me3 in all four groups

	H3K4me3			
	SCT			
	Control no vaccine	Control vaccine	COVID	S/P COVID
Number of values	16	13	15	16
Minimum	2	1	1	1
25% Percentile	6	5	2	2
Median	8	9	4	2
75% Percentile	12	10.5	6	4
Maximum	12	12	9	8
Range	10	11	8	7
95% CI of median				
Actual confidence level	97.87%	97.75%	96.48%	97.87%
Lower confidence limit	6	4	2	2
Upper confidence limit	12	12	6	4

	EVT			
	Control no vaccine	Control vaccine	COVID	S/P COVID
Number of values	16	13	15	16
Minimum	0	0	0	0
25% Percentile	0	0	0	0
Median	8	4	4	1.5
75% Percentile	12	8.5	12	2
Maximum	12	12	12	8
Range	12	12	12	8
95% CI of median				
Actual confidence level	97.87%	97.75%	96.48%	97.87%
Lower confidence limit	0	0	0	0
Upper confidence limit	12	9	12	2

	Decidual stroma			
	Control no vaccine	Control vaccine	COVID	S/P COVID
Number of values	16	13	15	16
Minimum	0	0	0	0
25% Percentile	0.25	0	0	0.25
Median	6	4	4	2
75% Percentile	10.5	8.5	6	5.5
Maximum	12	12	9	9
Range	12	12	9	9
95% CI of median				
Actual confidence level	97.87%	97.75%	96.48%	97.87%
Lower confidence limit	0	0	0	0
Upper confidence limit	12	9	6	6

**Fig. 2:**
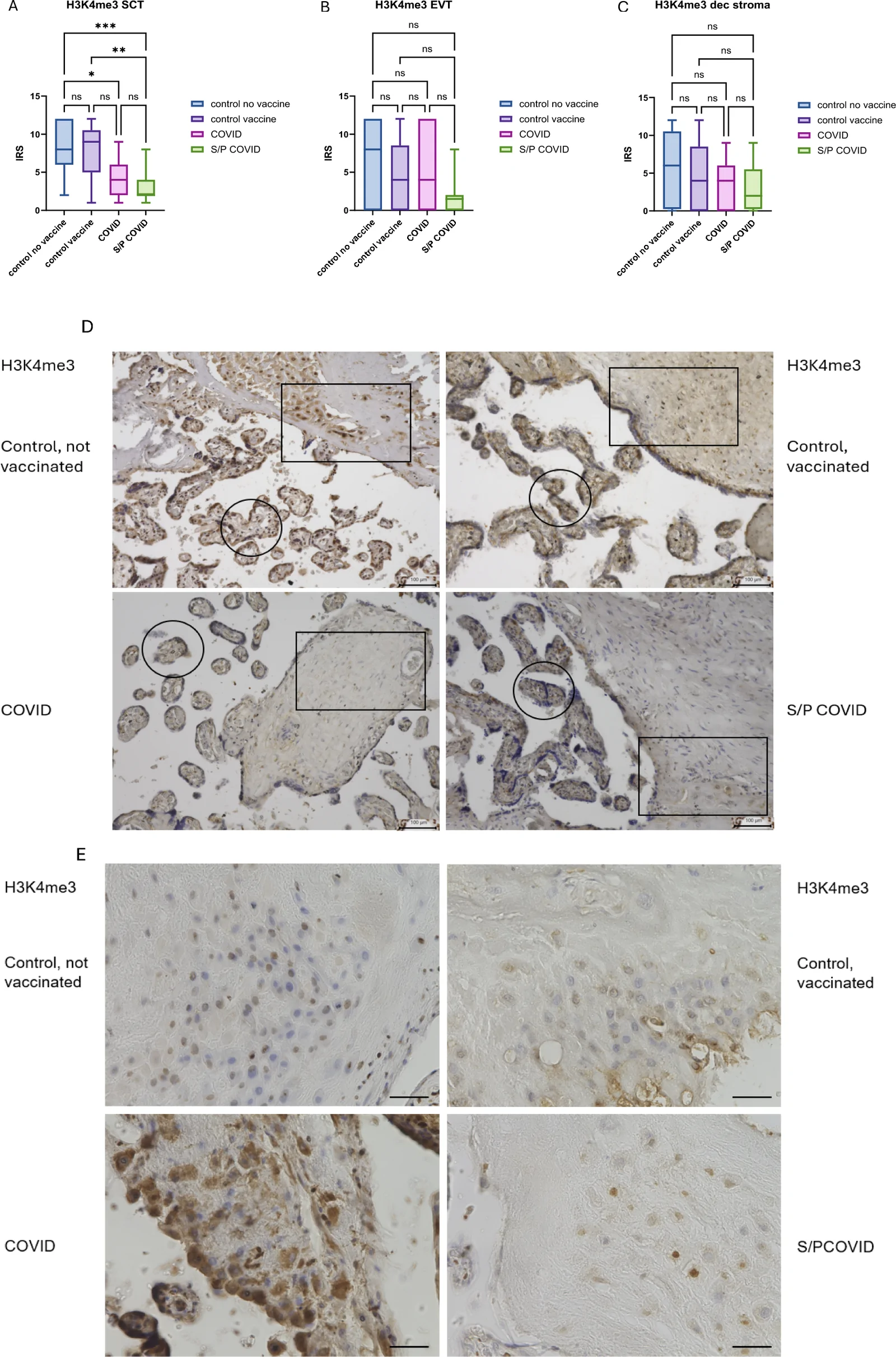
Staining results of trimethylated histone H3K4me3. Boxplots of IRS scores in the **A** syncytiotrophoblast (SCT) across the four groups, **B** EVT and **C** decidual stroma scored separately. Group differences were assessed by Kruskal–Wallis test followed by Dunn's post-hoc test with Bonferroni correction; significant pairwise comparisons are indicated (*p < 0.05; **p < 0.01; ***p < 0.001). **D** Representative immunohistochemical images of H3K4me3 in the SCT across all four groups; circles indicate SCT, squares indicate stem villi. **E** Representative immunohistochemical images of H3K4me3 in the decidua across all four groups. SCT represents the fetal villous compartment; decidua represents the maternal compartment of the placenta

In the SCT, median H3K4me3 IRS was lower in both the COVID group (median 4, IQR 2–6; p = 0.0308) and the S/P COVID group (median 2, IQR 2–4; p = 0.0002) compared to unvaccinated controls (median 8, IQR 6–12). The S/P COVID group also showed significantly lower values than vaccinated controls (median 9, IQR 5–10.5; *p* = 0.0029). No significant difference was observed between the two control groups (*p* > 0.999) or between the COVID and S/P COVID groups (*p* > 0.999). No significant differences in H3K4me3 IRS were observed in the EVT or decidual stromal compartments between groups. Representative staining images and boxplots are shown in Figure 2; complete IRS descriptive statistics for all compartments are provided in Table 2.

#### Acetylated histone H3K9ac in SARS-CoV-2-exposed placentas

H3K9ac expression was assessed using the same IHC approach described above. IRS scores were determined separately for the SCT, EVT, and decidual stromal cells. Because of non-normal distribution, results are reported as median (IQR). Group differences were assessed using the Kruskal–Wallis test followed by Dunn's post-hoc test with Bonferroni correction. Full descriptive statistics are shown in Table 3.

**Table 3:** Summary of IRS scores (median and IQR) of H3K9ac in all four groups

	H3K9ac			
	SCT			
	Control no vaccine	Control vaccine	COVID	S/P COVID
Number of values	16	13	15	16
Minimum	2	1	1	1
25% Percentile	4.5	3	4	3.25
Median	8	6	6	6
75% Percentile	12	8.5	12	9
Maximum	12	12	12	9
Range	10	11	11	8
95% CI of median				
Actual confidence level	97.87%	97.75%	96.48%	97.87%
Lower confidence limit	4	2	4	3
Upper confidence limit	12	9	12	9

	EVT			
	Control no vaccine	Control vaccine	COVID	S/P COVID
Number of values	16	13	15	16
Minimum	0	0	0	0
25% Percentile	0	0	1	0.25
Median	3.5	6	4	3
75% Percentile	11.25	8	6	6
Maximum	12	12	12	6
Range	12	12	12	6
95% CI of median				
Actual confidence level	97.87%	97.75%	96.48%	97.87%
Lower confidence limit	0	0	1	0
Upper confidence limit	12	8	6	6

	Decidual stroma			
	Control no vaccine	Control vaccine	COVID	S/P COVID
Number of values	16	13	15	16
Minimum	0	0	0	0
25% Percentile	0	0	2	4
Median	6	2	4	4
75% Percentile	11.25	7	9	6
Maximum	12	12	12	9
Range	12	12	12	9
95% CI of median				
Actual confidence level	97.87%	97.75%	96.48%	97.87%
Lower confidence limit	0	0	2	4
Upper confidence limit	12	8	9	6

In the SCT, median H3K9ac IRS was 8 (IQR 4.5–12) in unvaccinated controls, 6 (IQR 3–8.5) in vaccinated controls, 6 (IQR 4–12) in the COVID group, and 6 (IQR 3.25–9) in the S/P COVID group. Although median values were numerically lower in the infected and vaccinated groups compared to unvaccinated controls, the Kruskal–Wallis test followed by Dunn's post-hoc analysis revealed no statistically significant differences between any group pairs after correction for multiple comparisons (all adjusted *p* > 0.999). No significant differences in H3K9ac IRS were observed in the EVT or decidual stromal compartments either. Complete descriptive statistics for all compartments are provided in Table 3.

#### Correlation analysis

Spearman correlation analyses were performed across the entire cohort (n = 60) between IRS scores for H3K4me3 and H3K9ac in all three compartments and maternal age, gestational age at delivery, and birthweight. No significant differences were observed between placentas of male and female fetuses in any compartment (Table 4).

**Table 4:** Spearman correlation analysis between IRS scores and clinical variables (n = 60)

Comparison	Spearman r	p-value	Significance
Maternal age			
H3K4me3 SCT	0.081	0.539	ns
H3K4me3 decidual stroma	-0.052	0.695	ns
H3K4me3 EVT	-0.082	0.532	ns
H3K9ac SCT	0.031	0.817	ns
H3K9ac decidual stroma	-0.088	0.503	ns
H3K9ac EVT	-0.093	0.482	ns
Gestational age at delivery			
H3K4me3 SCT	0.053	0.689	ns
H3K4me3 Decidual stroma	0.256	0.048	*
H3K4me3 EVT	0.321	0.013	*
H3K9ac SCT	-0.040	0.763	ns
H3K9ac decidual stroma	0.230	0.077	ns
H3K9ac EVT	0.369	0.004	**
Birthweight (g)			
H3K4me3 SCT	0.021	0.875	ns
H3K4me3 decidual stroma	0.083	0.526	ns
H3K4me3 EVT	0.177	0.175	ns
H3K9ac SCT	0.007	0.958	ns
H3K9ac decidual stroma	0.234	0.072	ns
H3K9ac EVT	0.279	0.031	*

^*^ p < 0.05; ** p < 0.01; *ns* Not significant. *IRS* immunoreactive score, *SCT* syncytiotrophoblast, *EVT* extravillous trophoblast. Highlighted rows indicate statistically significant correlations

**Fig. 3:**
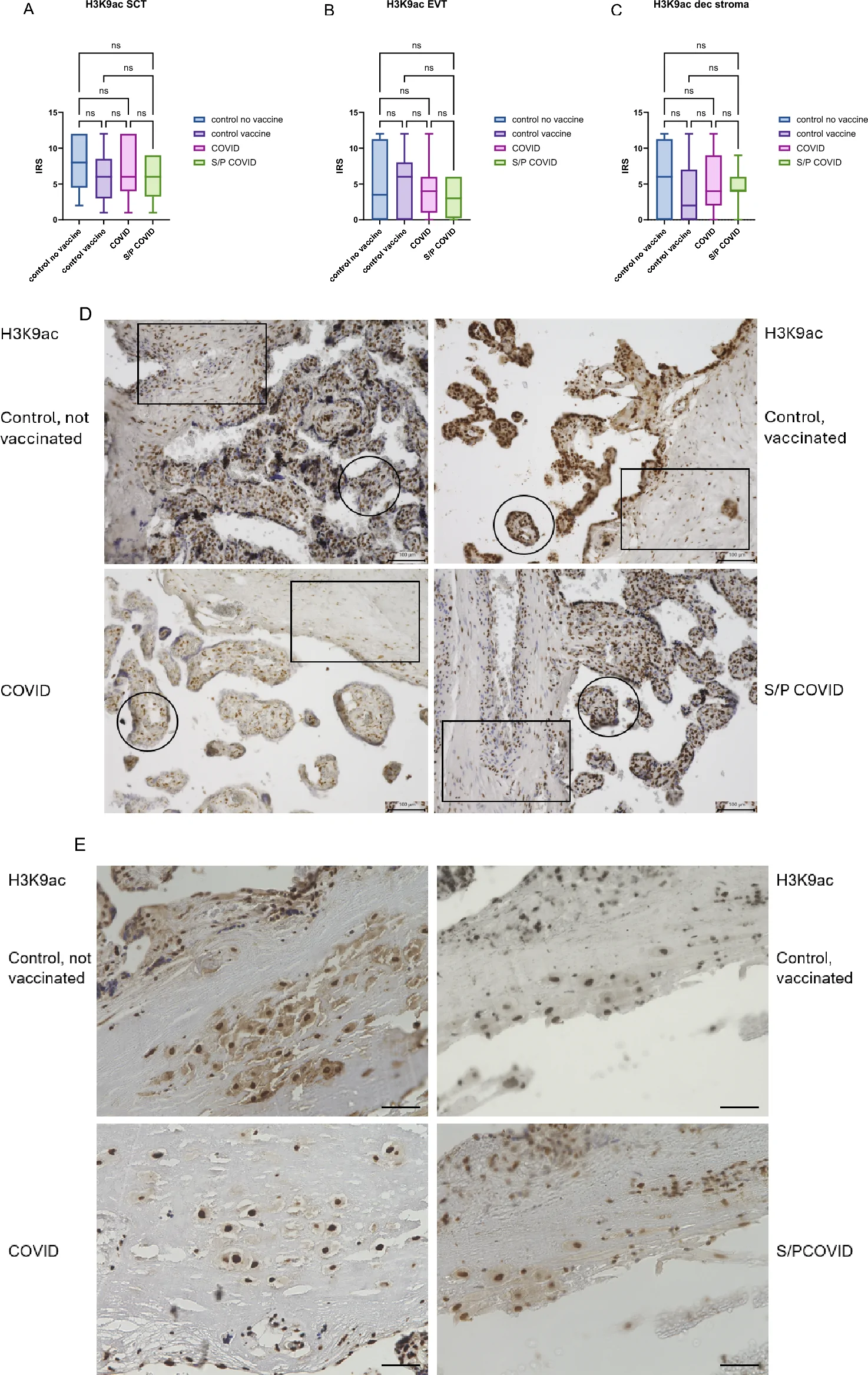
Staining results of acetylated histone H3K9ac. Boxplots of IRS scores in the **A** syncytiotrophoblast (SCT) across the four groups, **B** EVT and **C** decidual stroma scored separately. Group differences were assessed by Kruskal–Wallis test followed by Dunn's post-hoc test with Bonferroni correction; no pairwise comparisons reached statistical significance. **D** Representative immunohistochemical images of H3K9ac in the SCT across all four groups; circles indicate SCT, squares indicate stem villi. **E** Representative immunohistochemical images of H3K9ac in the decidua across all four groups. SCT represents the fetal villous compartment; decidua represents the maternal compartment of the placenta

Maternal age showed no significant correlation with any IRS score (all p > 0.48). Gestational age showed weak positive correlations with H3K4me3 IRS in the decidual stroma (r = 0.256, p = 0.048) and EVT (r = 0.321, p = 0.013), and with H3K9ac IRS in the EVT (r = 0.369, p = 0.004), suggesting that more preterm deliveries are modestly associated with lower decidual histone modification levels. Birthweight correlated weakly with H3K9ac EVT IRS (r = 0.279, p = 0.031). No significant correlations were identified between any clinical variable and SCT IRS for either marker. However, the absence of significant correlations cannot exclude confounding, and these findings should be interpreted as exploratory, unadjusted associations (Figure 3).

### Exploratory immunofluorescence staining

**Fig. 4:**
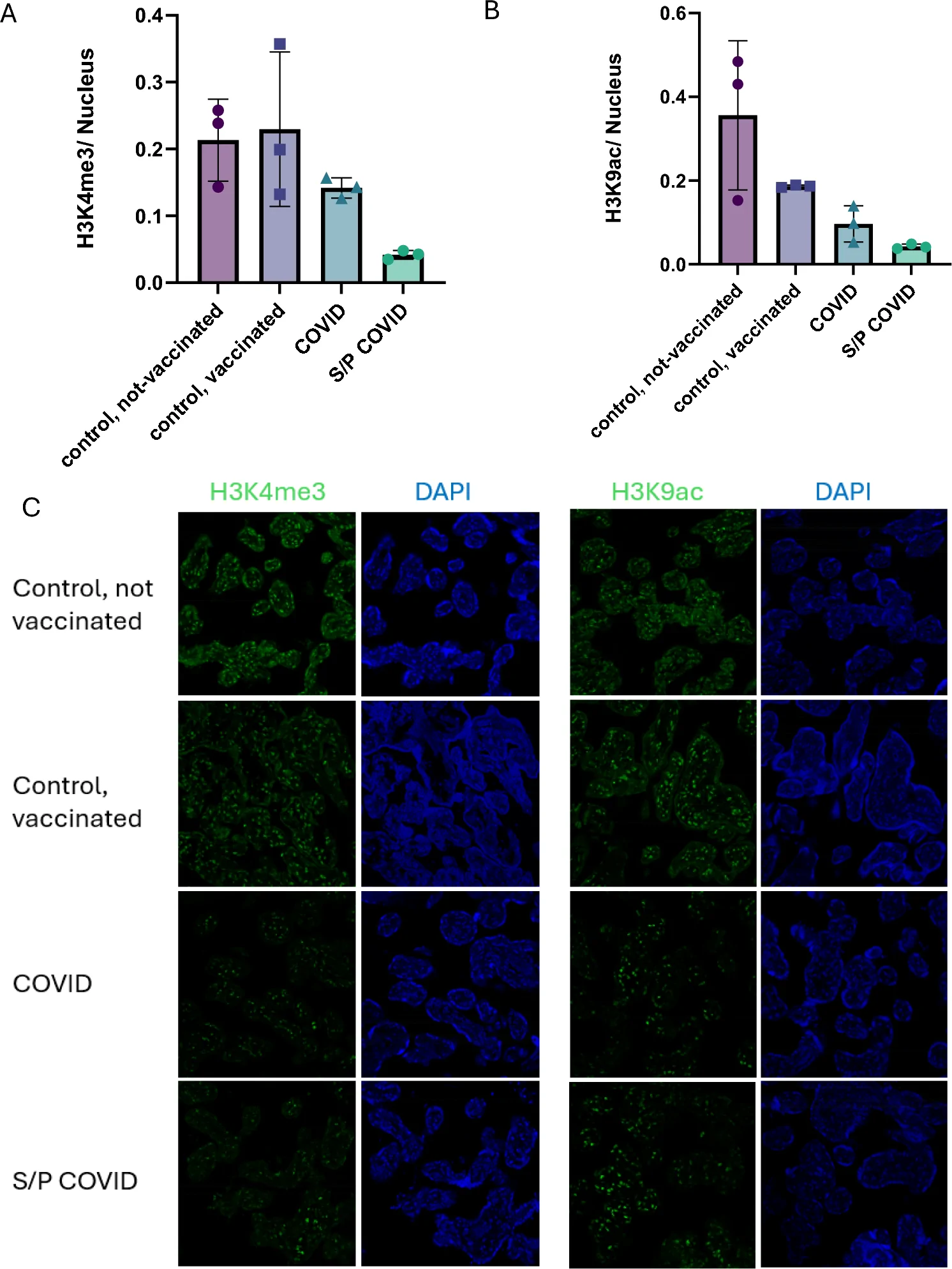
**A** Ratio of H3K4me3 to nucleus in placental tissue of all 4 groups. **B** Ratio of H3K9ac to nucleus in placental tissue of all 4 groups. **C** Representative immunofluorescence staining of H3K4me3 and H3K9ac (green) and DAPI (blue) in all 4 groups

Immunofluorescence imaging of H3K4me3 and H3K9ac was performed on three placental samples per group (unvaccinated control, vaccinated control, acute COVID-19 infection, and S/P infection) as a qualitative, exploratory complement to the IHC findings. No formal quantitative image-analysis protocol, blinding procedure, or statistical testing was applied; these data should be considered illustrative and not independently confirmatory. Both the acute COVID-19 and S/P infection groups showed a visually reduced ratio of histone modification signal intensity relative to nuclear (DAPI) staining, consistent with the IHC IRS findings (Figure 4).

## Discussion

This study was designed as a descriptive, hypothesis-generating analysis of global histone modification patterns in placental tissue from SARS-CoV-2-exposed pregnancies, rather than a locus-specific or functional epigenetic investigation.

Histone modifications are known to exert strong regulatory effects on transcriptional activity [22]. In a previous study, we demonstrated that H3K4me3 and H3K9ac levels are reduced in placentas from women with preeclampsia compared to healthy controls [19]. These modifications are commonly associated with transcriptionally permissive chromatin states and may reflect altered regulatory potential in placental tissue [19]. Histones facilitate the dense packing of DNA, while their N-terminal tails remain flexible and accessible for post-translational modifications [23]. Such modifications can influence chromatin structure and thereby modulate access of transcriptional machinery to DNA [24].

Various histone modifications have previously been implicated in pathological mechanisms relevant to placental dysfunction, including preeclampsia and viral infections [19]. Histone modifications have also been associated with regulatory regions of genes encoding growth factors critical for placental development, such as vascular endothelial growth factor (VEGF) and placental growth factor (PlGF) [25–27]. These regulatory mechanisms are particularly relevant for trophoblast invasion and migration, processes that are impaired in preeclampsia and have been suggested to be affected in the context of SARS-CoV-2 infection [16, 28, 29]. Dysregulation of these processes can contribute to insufficient placentation and adverse pregnancy outcomes, including intrauterine growth restriction, hypertension, and preterm birth [30].

In the present study, we investigated selected histone modifications in placentas from women with SARS-CoV-2 infection during pregnancy or at delivery, focusing on epigenetic alterations associated with maternal infection rather than direct viral infection of placental tissue. As analyses were performed on whole tissue sections, observed changes represent averaged signals across cell populations and may underestimate cell-type-specific effects. The primary finding of this study is a significant reduction in H3K4me3 IRS in the syncytiotrophoblast (SCT) of both the acute COVID group and the S/P COVID group compared to unvaccinated controls, with the most pronounced reduction observed in the S/P COVID group. No significant differences in H3K4me3 were observed in the EVT or decidual stromal compartments.For H3K9ac, no statistically significant differences were found between any groups in any compartment after correction for multiple comparisons, indicating that H3K9ac levels were not significantly altered in SARS-CoV-2-exposed placentas in this cohort.

H3K4me3 is typically enriched at promoters of actively transcribed genes [31]. In the present study, reduced H3K4me3 levels were observed in the SCT following SARS-CoV-2 exposure. However, as no gene expression profiling was performed, the observed differences cannot be interpreted as evidence of altered transcriptional activity. Viral infections, including SARS-CoV-2, have been reported to affect epigenetic regulatory mechanisms, potentially through inflammatory signaling and cellular stress responses [32, 33]. Thus, the observed differences in SCT H3K4me3 may reflect an epigenetic alteration associated with SARS-CoV-2 exposure, although the biological significance of this finding remains unclear and requires further investigation.

No significant differences in H3K4me3 or H3K9ac IRS were observed between placentas of male and female fetuses in any compartment. Spearman correlation analyses revealed no significant associations between SCT IRS scores for either marker and maternal age, gestational age, or birthweight. However, given the unadjusted, exploratory nature of these analyses, residual confounding by unmeasured clinical variables cannot be excluded.. However, gestational age showed weak but significant positive correlations with H3K4me3 IRS in the decidual stroma and EVT, and with H3K9ac IRS in the EVT, indicating that gestational age at delivery may act as a partial confounder for the decidual compartment findings. This is particularly relevant given that the acute COVID group had the lowest mean gestational age at delivery in this cohort.

Taken together, our findings demonstrate an association between SARS-CoV-2 infection during pregnancy and reduced H3K4me3 levels in the syncytiotrophoblast. These results provide a foundation for future studies integrating epigenetic analyses with bulk or spatial transcriptomic approaches to determine whether these epigenetic alterations translate into functional gene expression changes and impact placental physiology and pregnancy outcomes. These observations should not be interpreted as evidence against vaccination, as vaccinated controls did not show pathological placental histone modification patterns and vaccination serves a fundamentally different immunological role than active infection.

Several limitations of this study should be acknowledged. First, analyses were performed on whole placental tissue sections rather than isolated cell populations. We initially attempted fluorescence-activated cell sorting (FACS) to isolate specific cell types; however, despite multiple optimization efforts, this approach proved technically challenging due to the fragile nature and heterogeneity of placental samples, as well as low cell yield and viability after dissociation. Consequently, we proceeded with whole tissue sections, which limits cell-type-specific resolution of our findings. Second, locus-specific histone profiling at regulatory regions of functionally relevant genes such as VEGF and PlGF was not performed, and no gene expression data were generated; functional consequences of the observed H3K4me3 changes therefore remain unknown. Third, the western blot analysis was limited to n = 3 per group for the unvaccinated control, vaccinated control, and S/P COVID groups, and n = 2 for the acute COVID group, precluding formal statistical comparison; these data are exploratory only. Fourth, given the absence of serological testing, asymptomatic or undiagnosed prior SARS-CoV-2 infections cannot be fully excluded in the vaccinated control group. Fifth, the presence of placental pathologies including preeclampsia, placenta previa, and placenta accreta in a small number of cases distributed across groups represents a potential confounder that could not be formally addressed due to the limited sample sizes. To assess whether these pathologies influenced the primary findings, a sensitivity analysis was performed excluding the three affected cases. The pattern of significantly reduced H3K4me3 IRS in the SCT of SARS-CoV-2-exposed groups compared to unvaccinated controls was maintained and slightly strengthened after exclusion (control no vaccine vs. COVID: p = 0.0095; control no vaccine vs. S/P COVID: p = 0.0002), indicating that the main findings are robust to the removal of these cases. H3K9ac remained non-significant in the sensitivity analysis. Gestational age at delivery showed weak but significant correlations with histone modification levels in the decidual compartments, and given the lower mean gestational age in the acute COVID group, a partial contribution of gestational age to the decidual findings cannot be excluded. Finally, detailed gestational timing of prior SARS-CoV-2 infection and the interval between infection and delivery could not be retrieved for all cases; complete individual-level data are no longer available from medical records.

## Conclusions

In summary, this descriptive study demonstrates that SARS-CoV-2 infection during pregnancy is associated with significantly reduced H3K4me3 levels in the syncytiotrophoblast, with the most pronounced reduction observed in women with prior infection during gestation. These findings were robust to the exclusion of cases with additional placental pathologies. H3K9ac levels did not differ significantly between groups after correction for multiple comparisons. As no gene expression profiling was performed, conclusions regarding functional or transcriptional consequences of the observed H3K4me3 changes cannot be drawn. Future studies incorporating larger cohorts and integrative approaches, such as bulk or spatial transcriptomic analyses, will be necessary to determine whether these epigenetic alterations are linked to changes in placental gene expression and pregnancy outcomes.

## Data Availability

All data supporting the findings of this study are available within the paper and its Supplementary Information.

## Abbreviations

IHC: Immunohistochemical staining
IRS: Semi-quantitative immunoreactive score
SCT: Syncytiotrophoblast
EVT: Extravillous trophoblast
H3K4me3: Histone trimethylation at lysine 4
H3K9ac: Histone acetylation at lysine 9

## References

[1] da Silva RGL, Chammas R, Novaes HMD (2021) Rethinking approaches of science, technology, and innovation in healthcare during the COVID-19 pandemic: the challenge of translating knowledge infrastructures to public needs. Health Res Policy Syst 19:104. 10.1186/s12961-021-00760-8 PMC8293568 34289860

[2] Ochani R, Asad A, Yasmin F, Shaikh S, Khalid H, Batra S, Sohail MR, Mahmood SF, Ochani R, Hussham Arshad M, Kumar A, Surani, (2021) S COVID-19 pandemic: from origins to outcomes.a comprehensive review of viral pathogenesis, clinical manifestations, diagnostic evaluation, and management. Infez Med 29:20–36 (**PMID: 33664170**) 33664170

[3] Atzrodt CL, Maknojia I, McCarthy RDP, Oldfield TM, Po J, Ta KTL, Stepp HE, Clements TP (2020) A guide to COVID-19: a global pandemic caused by the novel coronavirus SARS-CoV-2. FEBS J 287:3633–3650. 10.1111/febs.15375 PMC7283703 32446285

[4] Hassan M, Zalkifal M, Wahab A, Afzal S, Rafique S, Shahid M, Khan MA, Ahmed N, Idrees M, Shahid AA (2021) Novel coronavirus: a review from origin to current status of therapeutic strategies. Crit Rev Eukaryot Gene Expr 31:21–34. 10.1615/CritRevEukaryotGeneExpr.2021038075 34369712

[5] Morelli AE, Sadovsky Y (2022) Extracellular vesicles and immune response during pregnancy: a balancing act. Immunol Rev 308:105–122. 10.1111/imr.13074 PMC9189022 35199366

[6] Abu-Raya B, Michalski C, Sadarangani M, Lavoie PM (2020) Maternal immunological adaptation during normal pregnancy. Front Immunol 11:575197. 10.3389/fimmu.2020.575197 PMC7579415 33133091

[7] Chen G, Liao Q, Ai J, Yang B, Bai H, Chen J, Liu F, Cao Y, Liu H, Li K (2021) Immune response to COVID-19 during pregnancy. Front Immunol 12:675476. 10.3389/fimmu.2021.675476.PMID:34012458;PMCID:PMC8126657 PMC8126657 34012458

[8] Juttukonda LJ, Wachman EM, Boateng J, Jain M, Benarroch Y, Taglauer ES (2022) Decidual immune response following COVID-19 during pregnancy varies by timing of maternal SARS-CoV-2 infection. J Reprod Immunol 151:103501. 10.1016/j.jri.2022.103501 PMC8867981 35231754

[9] Oliveira KF, Oliveira JF, Wernet M, Paschoini MC, Ruiz MT (2021) Vertical transmission and COVID-19: a scoping review. Rev Bras Enferm 74(Suppl. S1):e20200849. 10.1590/0034-7167-2020-0849 34037165

[10] Thapa B, Acharya S, Karki S (2021) Vertical transmission of COVID-19: a case report and review of literature. J Nepal Health Res Counc 19:203–205. 10.33314/jnhrc.v19i1.3312 33934161

[11] Al-Kuraishy HM, Al-Gareeb AI, Albezrah NKA, Bahaa HA, El-Bouseary MM, Alexiou A, Al-Ziyadi SH, Batiha GE (2023) Pregnancy and COVID-19: high or low risk of vertical transmission. Clin Exp Med 23:957–967. 10.1007/s10238-022-00907-z PMC9574177 36251144

[12] Schwartz DA, Mulkey SB, Roberts DJ (2023) SARS-CoV-2 placentitis, stillbirth, and maternal COVID-19 vaccination: clinical-pathologic correlations. Am J Obstet Gynecol 228(3):261–269. 10.1016/j.ajog.2022.10.001 PMC9554221 36243041

[13] Movahed F, Haji Hosseini F, Heidari A, Dehbozorgi M, Ataei M, Vahidi F, Abyaneh R, Bajelan B, Bakhtiyari M, Shafiee A (2025) COVID-19 vertical transmission from mothers to neonates: a systematic review and meta-analysis of 204 studies. J Infect Public Health 18(9):102825. 10.1016/j.jiph.2025.102825 40466390

[14] Wu S, Tang L, Liu Z, Wu M, He X, Shan S, Zhou Y, Lin K, Xu Q, Tan S, Zhang Z, Xu Y, Wang C, Zhu F, Wei Z, Jin L (2025) Presence of SARS-CoV-2 in fetal organs via intraamniotic infection. Nat Commun 16(1):10261. 10.1038/s41467-025-65131-1. (**PMID:41271675;PMCID:PMC12639148**) PMC12639148 41271675

[15] Bi S, Tu Z, Chen D, Zhang S (2023) Histone modifications in embryo implantation and placentation: insights from mouse models. Front Endocrinol 14:1229862. 10.3389/fendo.2023.1229862 PMC10436591 37600694

[16] Apicella C, Ruano CSM, Méhats C, Miralles F, Vaiman D (2019) The role of epigenetics in placental development and the etiology of preeclampsia. Int J Mol Sci 20:2837. 10.3390/ijms20112837 PMC6600551 31212604

[17] Io S, Kabata M, Iemura Y, Semi K, Morone N, Minagawa A et al (2021) Capturing human trophoblast development with naive pluripotent stem cells in vitro. Cell Stem Cell 28:1023–1039. 10.1016/j.stem.2021.03.013 33831365

[18] Zhu Y, Yu J, Niu Y, Sun Q (2024) Dynamic histone modifications in human trophoblast lineage differentiation. Sci Rep 14:4783. 10.1038/s41598-024-55189-0. (**PMID: 38402275**) PMC10894295 38402275

[19] Meister S, Hahn L, Beyer S, Kuhn C, Jegen M, von Schönfeldt V, Corradini S, Schulz C, Kolben TM, Hester A, Appelt T, Mahner S, Jeschke U, Kolben T (2021) Epigenetic modification via H3K4me3 and H3K9ac in human placenta is reduced in preeclampsia. J Reprod Immunol 145:103287. 10.1016/j.jri.2021.103287 33662848

[20] Meister S, Hahn L, Beyer S, Paul C, Mitter S, Kuhn C, von Schönfeldt V, Corradini S, Sudan K, Schulz C, Kolben TM, Mahner S, Jeschke U, Kolben T (2021) Regulation of epigenetic modifications in the placenta during preeclampsia: PPARγ influences H3K4me3 and H3K9ac in extravillous Trophoblast Cells. Int J Mol Sci 22:12469. 10.3390/ijms222212469.PMID:34830351;PMCID:PMC8622744 PMC8622744 34830351

[21] Hutter S, Knabl J, Andergassen U, Mayr D, Hofmann S, Kuhn C, Mahner S, Arck P, Jeschke U (2015) Fetal gender specific expression of tandem-repeat galectins in placental tissue from normally progressed human pregnancies and intrauterine growth restriction (IUGR). Placenta 36(12):1352–1361. 10.1016/j.placenta.2015.09.015 26462906

[22] Patsouras MD, Vlachoyiannopoulos PG (2019) Evidence of epigenetic alterations in thrombosis and coagulation: a systematic review. J Autoimmun 104:102347. 10.1016/j.jaut.2019.102347 31607428

[23] Iwasaki W, Miya Y, Horikoshi N, Osakabe A, Taguchi H, Tachiwana H, Shibata T, Kagawa W, Kurumizaka H (2013) Contribution of histone N-terminal tails to the structure and stability of nucleosomes. FEBS Open Bio 3:363–369. 10.1016/j.fob.2013.08.007 PMC3821030 24251097

[24] Taverna SD, Li H, Ruthenburg AJ, Allis CD, Patel DJ (2007) How chromatin-binding modules interpret histone modifications: lessons from professional pocket pickers. Nat Struct Mol Biol 14:1025–1040. 10.1038/nsmb1338 PMC4691843 17984965

[25] Rahat B, Najar RA, Hamid A, Bagga R, Kaur J (2017) The role of aberrant methylation of trophoblastic stem cell origin in the pathogenesis and diagnosis of placental disorders. Prenat Diagn 37:133–143. 10.1002/pd.4974. (**PMID: 27885689**) 27885689

[26] Tudisco L, Della Ragione F, Tarallo V, Apicella I, D’Esposito M, Matarazzo MR, De Falco S (2014) Epigenetic control of hypoxia inducible factor-1α-dependent expression of placental growth factor in hypoxic conditions. Epigenetics 9:600–610. 10.4161/epi.27835.PMID:24504136;PMCID:PMC4121370 PMC4121370 24504136

[27] Gallardo V, González M, Toledo F, Sobrevia L (2020) Role of heme oxygenase 1 and human chorionic gonadotropin in pregnancy associated diseases. Biochimica et Biophysica Acta (BBA) - Molecular Basis of Disease 1866:165522. 10.1016/j.bbadis.2019.07.016 31376481

[28] Kamrani A, Alipourfard I, Ahmadi-Khiavi H, Yousefi M, Rostamzadeh D, Izadi M, Ahmadi M (2019) The role of epigenetic changes in preeclampsia. BioFactors 45:712–724. 10.1002/biof.1542 31343798

[29] Staff AC, Dechend R, Pijnenborg R (2010) Learning from the placenta: acute atherosis and vascular remodeling in preeclampsia-novel aspects for atherosclerosis and future cardiovascular health. Hypertension 56:1026–1034. 10.1161/HYPERTENSIONAHA.110.157743. (**PMID: 20956732**) 20956732

[30] Moser G, Windsperger K, Pollheimer J, de Sousa Lopes SC, Huppertz B (2018) Human trophoblast invasion: new and unexpected routes and functions. Histochem Cell Biol 150:361–370. 10.1007/s00418-018-1699-0 PMC6153604 30046889

[31] Wang H, Fan Z, Shliaha PV, Miele M, Hendrickson RC, Jiang X, Helin K (2023) H3K4me3 regulates RNA polymerase II promoter-proximal pause-release. Nature 615:339–348. 10.1038/s41586-023-05780-8 PMC9995272 36859550

[32] Gandhi S, Mitterhoff R, Rapoport R, Farago M, Greenberg A, Hodge L, Eden S, Benner C, Goren A, Simon I (2022) Mitotic H3K9ac is controlled by phase-specific activity of HDAC2, HDAC3, and SIRT1. Life Sci Alliance 5:e202201433. 10.26508/lsa.202201433 PMC9389593 35981887

[33] Sen R, Garbati M, Bryant K, Lu Y (2021) Epigenetic mechanisms influencing COVID-19. Genome 64:372–385. 10.1139/gen-2020-0135 33395363

